# Phenotypic transitions enacted by simulated microgravity do not alter coherence in gene transcription profile

**DOI:** 10.1038/s41526-019-0088-x

**Published:** 2019-11-21

**Authors:** Agnese Po, Alessandro Giuliani, Maria Grazia Masiello, Alessandra Cucina, Angela Catizone, Giulia Ricci, Martina Chiacchiarini, Marco Tafani, Elisabetta Ferretti, Mariano Bizzarri

**Affiliations:** 1 Department of Molecular Medicine, Sapienza University, Rome, Italy; 2 Environment and Health Department, Istituto Superiore di Sanità, Rome, Italy; 3 Department of Surgery “Pietro Valdoni”, Sapienza University, Rome, Italy; 4 Azienda Policlinico Umberto I, Rome, Italy; 5 Department of Anatomy, Histology, Forensic-Medicine and Orthopedics, Sapienza University, Rome, Italy; 6 Department of Experimental Medicine, Università degli Studi della Campania “Luigi Vanvitelli”, Naples, Italy; 7 Department of Experimental Medicine, Sapienza University, Rome, Italy; 8 Systems Biology Group Lab, Sapienza University, Rome, Italy

**Keywords:** Biophysics, Systems biology

## Abstract

Cells in simulated microgravity undergo a reversible morphology switch, causing the appearance of two distinct phenotypes. Despite the dramatic splitting into an adherent-fusiform and a floating-spherical population, when looking at the gene-expression phase space, cell transition ends up in a largely invariant gene transcription profile characterized by only mild modifications in the respective Pearson’s correlation coefficients. Functional changes among the different phenotypes emerging in simulated microgravity using random positioning machine are adaptive modifications—as cells promptly recover their native phenotype when placed again into normal gravity—and do not alter the internal gene coherence. However, biophysical constraints are required to drive phenotypic commitment in an appropriate way, compatible with physiological requirements, given that absence of gravity foster cells to oscillate between different attractor states, thus preventing them to acquire a exclusive phenotype. This is a proof-of-concept of the adaptive properties of gene-expression networks supporting very different phenotypes by coordinated ‘profile preserving’ modifications.

## Introduction

Living organisms on the surface of the Earth experience dramatic changes when the gravity environment changes from Earth gravity (1*g*) to microgravity in space. Such changes cover a wide range of biological consequences ranging from microbial growth to immune functions in astronauts.^1^ As evidenced by microarray studies, these changes are associated with modifications in gene expression, both in real and simulated microgravity.^2–4^ As a result, understanding how and why cells and tissues modify their behaviour and morphology in absence of gravity recently became a new paradigm for widening and deepening our knowledge of the role of constraints in Biology.^5^

It is worth noting that human cell types cultured in microgravity undergo dramatic morphology changes, leading to two alternative phenotypes: an ‘adherent’ and a ‘floating cell clumps’ one, simultaneously present in the same culture.^6–8^ This is a reversible process: when a ‘clumps-organoid’ population is seeded in normal gravity, it goes back to the usual phenotype and, when reseeded in microgravity condition, it gives rise to the two above-mentioned phenotypes. A similar behaviour is observed when the experiment is reiterated starting with cells obtained from the adherent phenotype. To the best of our knowledge, only one study has investigated the differences in gene expression between the two morphologic phenotypes emerging in microgravity.^9^ Yet, even in this case, the overall coherence (i.e. the stability of the autocorrelation values among gene expressions along the transition through different phenotypic states) in both phenotypes has never been performed up to now.

Here we are focusing on two alternative hypotheses about the relation between morphology/functionality and gene expression. (a) The relaxation of gravity constraint allows the appearance of two alternative morphological quasi-equilibrium states, while the observed gene expression changes are the consequence of a true critical transition, implying a complete rewiring of the gene activity pattern, thus allowing the system to log into a very new attractor state. (b) The two morphological states can be considered as proper ‘sub-attractors’ of the shared cell-kind attractor in the multidimensional gene-expression space, and thus are fully adaptive to sustain the two alternative phenotypes. This implies the gene-expression changes happen in a coordinate manner across the genome, with only minor variations, preserving overall coherence, i.e. showing high autocorrelation values along the different sub-state the system is travelling.

The experimental model was designed to discriminate between the above two hypotheses.

Herein we use coarse-grain statistical metrics for estimating the behaviour of a gene ensemble, adopting a statistical mechanics-inspired model of biological regulation.^10^ Accordingly, whole-genome expression is ruled by self-organization processes, as described by the Self Organized Criticality (SOC) theory.^11^

Briefly, SOC considers a cell-fate decision-making model in which the cell population can select among diverse cell-fate options that are already expressed by a few cell clusters, each one characterized by the expression of a well-defined transcriptional “program”. As the system approaches a critical state—in which gene-expression stability is challenged and fluctuation rate of several biological activities are increased—the interplay between intracellular factors and environmental constraints can drive the overall system in selecting only one among the available transcriptional programs. Then, a cell-fate gene module is selectively amplified, allowing the system acquiring stability and coherence, as measured by computing Pearson correlation coefficient.^12–14^ When the system undergoes a transition toward another state, this correlation changes in a relevant manner.^15^

We set out in investigating changes in correlation values between gene-expression profiles to check for a ‘gene-expression profile counterpart’ of the morphological changes induced by simulated microgravity. We must demonstrate that the autocorrelation is detectable given we select a sufficiently wide range of mean expressions of any choice of probe genes, instead of analysing the overall transcriptome landscape, as already reported in greater details.^11,16,17^ Here we focused on an appropriate set of 26 gene expression data. These 26 genes—independent of their biological function they fulfil—were chosen with a sufficiently diverse average expression range of variation (d = 0.60). We selected genes relative to a coherent network that, as expected, gave rise to very high average between genes correlation (r = 0.834, SD = 0.07). This between genes strict correlation is instrumental for dealing with eventual missing values (Supplementary Methods). The quasi-stability condition would be mirrored by the observation of near-to-unity autocorrelation values across different times for gene-expression profiles in the three experimental conditions: OG (cells On Ground), RPMAD (Adherent Cells obtained in simulated microgravity by Random Positioning Machine) and RPMCLUM (Random Machine Positioning Clump cells). Deviation from the stability have been quantitated by considering Euclidean and Angular distances from reference on ground state. The Euclidean distance measures how the expression gene profiles differ in magnitude among the different samples, e.g. to what extent the same genes are differently expressed (Supplementary Fig. 3). On the contrary, cosine (angular) distances correspond to the angle between two vectors. Pearson correlation coefficient permits us to measure such distance as it corresponds to the cosine of the angle between the two analysed variable vectors (in our case the gene-expression profiles). The angle distance distinguishes how gene-expression pattern differs in terms of relation among different gene expression, thus recognizing a qualitative difference in their expression pattern. In practice, if we have three genes with expression values 10, 20, 30 in sample A and 20, 40, 60 in sample B, they have non-zero Euclidean distance (d(AB) = 37.4). On the contrary, they have a zero angular distance (the two A and B vectors have a Pearson correlation r = 1, given the proportions among their components do not differ), being Pearson r the cosine of the angle between the two vectors that is to say the two vectors are parallel (zero angle).^18^

## Results

### MCF7 cells in simulated microgravity undergo a morphological and phenotypic transition

#### Morphological changes

**Fig. 1:**
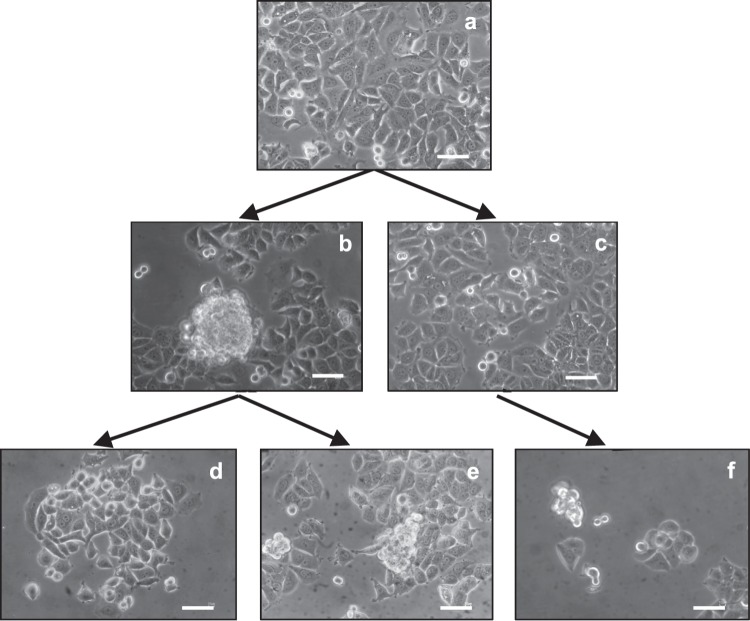
Morphological changes in cells exposed to different gravity conditions. MCF7 cells cultured under static 1g-conditions grew as a normal 2D monolayer **a**. MCF7 cells in microgravity resulted partitioned into two phenotypes. The first, represented by floating-clump (RPMCLUM) cells, and the second constituted by adherent cells (RPMAD) (**b**, **c**). Both phenotypes revert to the native morphology when they are reseeded in normal gravity, independently from the time they have spent in microgravity. In panel **d** it is shown how cells growing in microgravity for 24 hours recover their native phenotype when replaced in normal gravity for 6 h. When the two cell clusters previously obtained during a first-course culture in weightlessness are isolated, and then again reseeded in the same microgravity field, two distinct phenotypes emerge once more from each cell phenotype (**e**, **f**). Scale bar: 50 μm.

Phase contrast microscopy showed that MCF7 cells cultured under static 1g-conditions (next to RPM) grew as a normal 2D monolayer (Fig. 1a), displaying the usual flat shaped morphology while adhering to the flask bottom. However, after a 1 h exposure to simulated microgravity, MCF7 cells resulted divided almost equally (49% versus 50%) into two phenotypes. The first, represented by floating-clump (RPMCLUM) cells, constituted by small, rounded cells growing as 3D organoid-like structure; the other one, comprising adherent cells (RPMAD), growing in a unidimensional monolayer. After exposure to simulated microgravity, the two distinct clusters of cells (Fig. 1b, c) demonstrated stability over a period of 72 h (data not shown). The relative proportion of the two morphological clusters remains invariant for the full period of observation, suggesting the intrinsic dynamics between the two populations is operating close to the equilibrium. Both phenotypes revert to the native morphology when reseed in normal gravity, independently from the time they have spent in simulated microgravity (from 1 to 72 h). As an example, Fig. 1d shows how cells growing in simulated microgravity for 24 h recover their native phenotype when placed back in normal gravity for 6 h. As observed, when two cell clusters that have been exposed to simulated microgravity are isolated and reseeded in the same simulated microgravity field, two distinct phenotypes emerge once more from each cell cluster (i.e. adherent and floating) (Fig. 1e,f). When the same cell clusters were seeded again in normal gravity, they recovered their normal morphological phenotype.

To appreciate the quantitative aspects of such morphological changes, we investigated several parameters after 24 h of exposure. As reported in Table 1, RPMAD and RPMCLUM differed significantly in roundness, solidity and fractal dimension (FD). These differences persisted when RPMCLUM cells were compared with OG cells, while RPMAD cells showed only minor changes relative to the OG group. Even immortalized, normal breast cells (MCF10A), when exposed to simulated microgravity, resulted to be partitioned into two different morphological clusters with distinctive quantitative morphological and phenotypic traits (paper submitted).

**Table 1:** Quantitative morphological descriptors of cell shape in normal gravity (OG) and after 24 h of simulated microgravity

	Roundness	±SD	Solidity	±SD	Fractal dimension (FD)	±SD
OG	0.646	±0.151	0.802	±0.101	1.1560	±0.0356
RPMAD	0.667	±0.139	0.844	±0.130^a^	1.1381	±0.0230
RPMCLUM	0.783	±0.111^b^	0.921	±0.034^b^	1.1275	±0.1017^a^

^a^*p* < 0.01; ^b^*p* < 0.005

#### Proliferation and apoptosis

**Fig. 2:**
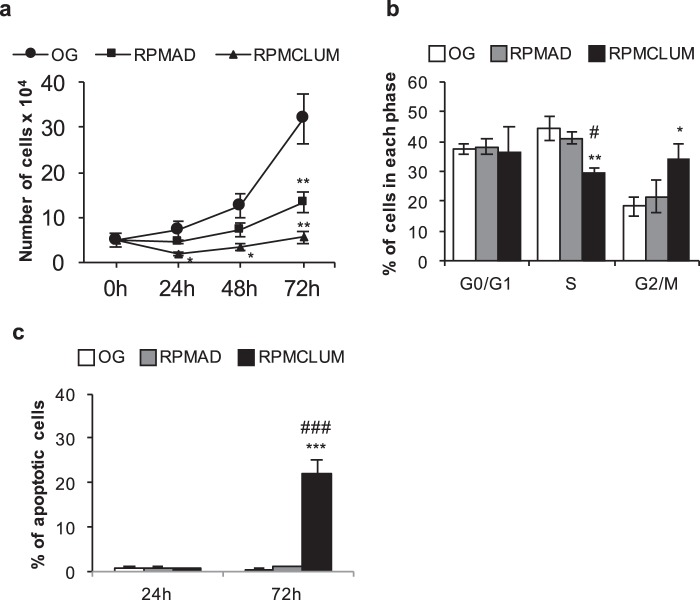
Proliferation, cell cycle and apoptosis. Proliferation rate was slightly reduced in both RPMAD and RPMCLUM **a**, during the first 24 h of microgravity conditioning. However, after 72 h, proliferation starts again in both phenotypes. Overall, both phenotypes show a reduced proliferation rate with respect to OG values. Distribution of cells within the cell cycle is shown in **b**. RPMCLUM cells in S-phase were significantly decreased, whereas cells in the G2/M phase increased up to 2-fold. On the contrary, RPMAD cells were almost unaffected by simulated microgravity. Apoptosis rate was unaffected in both cell clusters during the first 24 h, while a significant increase in programmed cell death emerges at 72 hours, only in the RPMCLUM group **c**.

Almost all studies carried out on cells cultured in weightlessness have reported small, albeit significant, changes in growth and apoptosis rate. To investigate if such condition represents a true critical factor threatening cell viability in space we investigated cell cycle, proliferation and apoptosis after 24 h of simulated microgravity. As expected, proliferation rate was slightly reduced in both RPMAD and RPMCLUM, with the lowest growth rate observed in the latter cluster (Fig. 2a). After 72 h, proliferation starts again in both RPMAD and RPMCLUM cells, with RPMCLUM displaying a significantly reduced growth rate compared to RPMAD cells. However, the proliferation rate of both phenotypes was reduced relative to the OG cells. In order to correlate these findings with the cell cycle, cytofluorimetric analysis was performed. As expected, MCF7 cells subjected to microgravity displayed appreciable modifications of their cell cycle when compared to OG condition (Fig. 2b). RPMCLUM cells in S-phase were significantly decreased, whereas cells in G2/M phase increase up to 2-fold. On the contrary, RPMAD cells were almost unaffected by simulated microgravity, even if a trend in reduction of cells into the S-phase can be noticed. These findings indicate that MCF7 cells growing in simulated microgravity slow-down their replication rate. Additionally, apoptosis rate was unaffected in both cell clusters during the first 24 h, while a significant increase in programmed cell death emerges at 72 h, only in the RPMCLUM group (Fig. 2c). To investigate whether the increase of apoptosis displayed by clump cells might be due to the lack of adhesion or the lack of nutrients in the centre of the clump, we evaluated apoptosis in MCF7 cells grown for up to 72 h in normal gravity and in non-adherent conditions. Cells grew in clumps and did not show significantly enhanced apoptosis respect to cells growing in monolayer (OG) (Supplementary Fig. 1).

#### Cytoskeleton

**Fig. 3:**
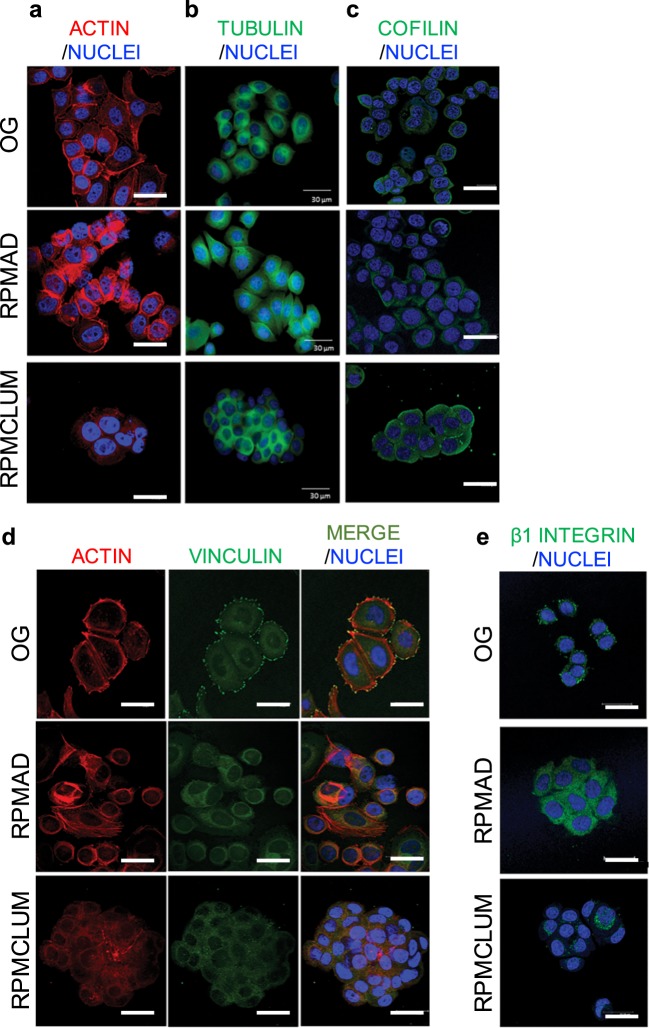
Cytoskeleton proteins in cells exposed to microgravity. Panels **a**, **b** show F-actin **a** and tubulin **b** respectively in MCF7 cells on ground and in RPM. In OG cells the network of cytosolic F-actin appears well organized in bundles associated with the cell plasma membrane. In RPMAD cells, stress fibers are less evident and F-actin bundles appeared mostly localized at the cell border. In floating cell clumps, the actin meshwork loses its organization and actin filaments looked short, fragmented and spreading over the cytosol. About the tubulin organization, we observed that the microtubule-organizing centre (MTOC) near the nucleus in OG cultured cells, disappears in both RPMAD and RPMCLUM **b**. However, in RPMAD samples microtubules are still identifiable, while in RPMCLUM samples tubulin meshwork was completely disrupted, and tubulin appeared almost completely aggregated around the nucleus without any polarization. Panel **c** shows cofilin distribution. Cofilin was dispersed in the whole cell body with a visible accumulation in the cytosol of OG and RMPAD cells; instead, in RPMCLUM cells, an impressive, dense accumulation of cofilin was observed under the cortical ring of the cellular membrane. Vinculin distribution is reported in panel **d**. Vinculin decreases in RPMAD group, and especially in both the cytosol and the membrane of RPMCLUM cells. Reduction of vinculin is accompanied by a reduced amount of stress fibers, formation of fewer focal adhesions, and inhibition of lamellipodia extension. Integrin distribution is also significantly impaired by microgravity **e**. An intense deposition of β1-integrin at the cell membrane in RPMAD cells is recorded, while in RPMCLUM cells β1-integrin almost completely disappears. Scale bars: 30 μm.

To get a deeper understanding of the cytoskeletal changes supporting morphological modifications, cytoskeleton proteins and their organization were investigated. After 24 h of simulated microgravity, both RPMAD and RPMCLUM cells showed a large rearrangement of F-actin and microtubules when compared to on ground control cells. In detail, in OG cells the network of cytosolic F-actin appears well organized in bundles associated to the cell plasma membrane, with a well-defined net of stress fibers recognizable in the cytosol (Fig. 3a). In RPMAD cells, stress fibers are less evident and F-actin bundles appeared mostly localized at the cell border (Fig. 3a, central panel). In floating cell clumps (RPMCLUM), the actin meshwork loses its organization, and actin filaments appeared short, fragmented and spreading over the cytosol. Moreover, stress fibers are almost undetectable in RPMCLUM samples (Fig. 3a bottom panel).

We observed also the disappearance of the polarized fluorescence associated to microtubule-organizing center (MTOC) near the nucleus in both RPMAD and RPMCLUM samples compared with OG cultured cells (Fig. 3b). Yet, in RPMAD samples microtubules are still identifiable, even if they do not emerge apparently from the MTOC, but from the whole perinuclear space. Instead, in RPMCLUM samples tubulin meshwork was disrupted, and tubulin appeared almost completely aggregated around the nucleus without any polarization. These findings were almost superimposable to those obtained by Grimm’s team in thyroid cells.^9^

To obtain some insights into the motile phenotype of the three cell clusters, we decided to evaluate cofilin and vinculin distribution through immunofluorescence.

Cofilin is an essential protein that controls actin depolymerization at the leading edge of motile cells, particularly by increasing the speed of actin treadmilling process. We found that cofilin was dispersed in the whole cell body with a visible accumulation in the cytosol of OG and RMPAD cells (Fig. 3c). Instead, in RPMCLUM cells, an impressive, dense accumulation of cofilin was observed in the most dynamic cellular area, i.e. under the cellular membrane known as ‘cortical ring’. These results suggest that the two phenotypes likely differ in their migratory potential, as evidenced by studies in which cofilin distribution inside the cells has been correlated with their respective motility.^19^

Vinculin is a cytoskeletal protein associated with cell-matrix and cell–cell junctions required for anchoring F-actin to the membrane and thus mechanically stabilizing the cell shape.^20^ Notably, the distribution pattern of vinculin is severely affected by simulated microgravity. Vinculin decreases in RPMAD group and significantly diminishes in both the cytosol and the membrane of RPMCLUM cells, with disappearance of vinculin aggregates at the focal adhesion foci (Fig. 3d). Reduction of vinculin is accompanied by reduced amount of stress fiber, formation of fewer focal adhesions, and inhibition of lamellipodia extension. Lack of vinculin may decrease cell adhesion by inhibiting focal adhesion assembly, thus preventing actin polymerization. Additionally, reduced vinculin levels may in principle counteract apoptosis, through both an ERK-dependent and independent pathway.^21^

Integrin distribution is dramatically impaired by simulated microgravity (Fig. 3e). While immunostaining shows an intense deposition of β1-integrin at the cell membrane in RPMAD cells, even significantly higher than that recorded in OG, in RPMCLUM cells, which grow free-floating, β1-integrin almost completely disappears as previously recorded in thyroid cells exposed to microgravity.^22^ Depletion of β1-integrin in RPMCLUM cells may have contributed to the enhanced apoptosis rate observed in this condition, given that it is well established that survival of many cell types requires integrin-mediated adhesion to extracellular matrix proteins.^23^

#### Genes

**Fig. 4:**
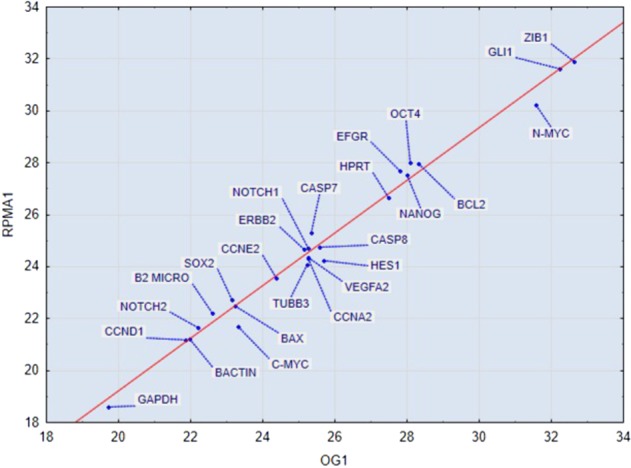
Pairwise correlation between gene-expression profile in cells growing at OG and RPMAD at 1 h. The correlation coefficients are near to unity, pointing to a very strong invariance of gene-expression profiles despite the dramatic phenotypic changes. Vector points correspond to gene-expression values, and the axes refer to the different conditions (microgravity exposure and on ground, after 1 h of conditioning).

We examined transcription data from a set of genes chosen with a sufficiently diverse average expression range of variation in order to highlight their attractor-like behaviour. Expression data are listed in Supplementary Table 1 and shown in Supplementary Fig. 2. Only changes higher than two-fold the control values were considered as significant. Genes were distributed in different classes, according to the function they are usually considered to belong. (1) Reprogramming genes: Sox2, Nanog, Oct4. Only Nanog and Sox2 shown to be significantly up regulated after 24 h in RPMAD cells. (2) Epithelial-Mesenchymal Transition-related genes. Zeb1 and VEGFA2 were significantly augmented at 24 h only in RPMAD cells. (3) Apoptotic pathways: caspase 7,8, Bax and Bcl2. Slight changes were observed in both caspases, while the apoptotic index BAX/Bcl2 increases in a significant manner at 24 h only in RPMCLUM cells, coherently with increased apoptosis in RPMCLUM, as shown in Fig. 4d. (4) Multidrug Resistance, Hedgehog and Notch Genes: c-Myc, Notch, Hes1. Notch2, Hes1 and N-Myc were significantly down regulated in RPMCLUM cells at 24 h. (5) Cell cycle: Cyclin A and E were up regulated after 24 h in RPMAD cells. Overall, these fluctuations have no clear meaning when matched with the biological functions displayed by cell samples. For instance, although RPMAD cells showed only minor morphological changes, they demonstrated a significant increase in EMT and reprogramming related genes.

### Morphological phenotype transitions of MCF7 are quasi-stable attactor states and cells preserve their gene-expression pattern

The expression vectors for each experimental sample have been calculated in normal gravity (OG) and in simulated microgravity conditions (RPMAD and RPMCLUM), at different time points (1, 2, 6 and 24 h), in order to obtain the respective Pearson correlation value (Table 2).

**Table 2:** Pearson correlation coefficients between gene-expression profiles

	OG1	OG2	OG6	OG24	RPMAD1	RPMAD2	RPMAD6	RPMAD24	RPMCL1	RPMCL2	RPMCL6	RPMCL24
OG1	1	0.99545	0.98874	0.98498	0.99269	0.98903	0.98821	0.95613	0.98509	0.97551	0.98656	0.99303
		<0.0001	<0.0001	<0.0001	<0.0001	<0.0001	<0.0001	<0.0001	<0.0001	<0.0001	<0.0001	<0.0001
OG2		1	0.99440	0.98840	0.99121	0.99255	0.99491	0.97022	0.98445	0.97318	0.98954	0.99486
			<0.0001	<0.0001	<0.0001	<0.0001	<0.0001	<0.0001	<0.0001	<0.0001	<0.0001	<0.0001
OG6			1	0.98094	0.98950	0.98593	0.99585	0.95731	0.98667	0.97756	0.99301	0.99213
				<0.0001	<0.0001	<0.0001	<0.0001	<0.0001	<0.0001	<0.0001	<0.0001	<0.0001
OG24				1	0.98189	0.98619	0.97960	0.95514	0.96898	0.95926	0.97239	0.98260
					<0.0001	<0.0001	<0.0001	<0.0001	<0.0001	<0.0001	<0.0001	<0.0001
RPMAD1					1	0.98925	0.98623	0.95151	0.99304	0.98407	0.98698	0.99201
						<0.0001	<0.0001	<0.0001	<0.0001	<0.0001	<0.0001	<0.0001
RPMAD2						1	0.98654	0.97274	0.97285	0.95789	0.97839	0.98896
							<0.0001	<0.0001	<0.0001	<0.0001	<0.0001	<0.0001
RPMAD6							1	0.96505	0.98514	0.97574	0.99355	0.99097
								<0.0001	<0.0001	<0.0001	<0.0001	<0.0001
RPMAD24								1	0.93787	0.92226	0.95578	0.96736
									<0.0001	<0.0001	<0.0001	<0.0001
RPMCL1									1	0.99544	0.98875	0.98426
										<0.0001	<0.0001	<0.0001
RPMCL2										1	0.98121	0.97361
											<0.0001	<0.0001
RPMCL6											1	0.99361
												<0.0001
RPMCL24												1

The pairwise between profiles Pearson correlation coefficients are reported together with their statistical significance values

The between sample average correlation coefficient is near to unity (r = 0.986, Std Dev.= 0.091, ranging from 0.975 to 0.993) for all the examined conditions, which points to a very strict global integration of cell populations in terms of gene expression. These results show that cells retain their basic gene-expression pattern—despite the recorded morphological changes—given the near to unity values of gene-expression profile correlations. As expected, adherent population (RPMAD1) gene-expression profile is more similar to On Ground (OG1) population than Clumps (RPMCLUM1) state (r(OG, RPMAD) = 0.993; r(OG, RPMCLUM) = 0.985). On the other hand, the slightly higher correlation value between RPMAD and RPMCLUM samples (r = 0.993), when compared to the correlation with OG1, provides support to the hypothesis that the common exposition to RPM condition has a peculiar ‘gene expression’ signature. These findings are consistent with the existence of a single cell-kind acting as an attractor state. To highlight such behaviour, in Fig. 4 we report the correlation between OG and RPMAD, both considered after 1 h of conditioning in both conditions.

**Fig. 5:**
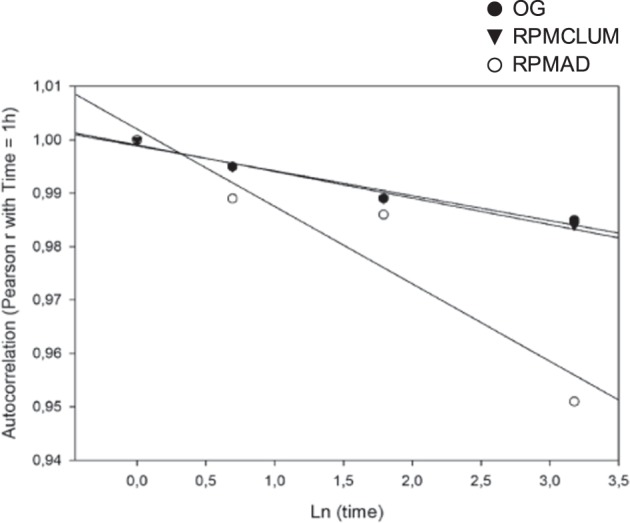
Autocorrelation time course of each cell population. The time course of the three OG, RPMAD, and RPCLUM conditions are reported in the figure in a plot having as abscissa the natural logarithm of time and as ordinate the autocorrelation with initial state relative to each condition. All the three conditions show a slight decrease (albeit highly significant, *p* < 0.001) in their autocorrelation along time, but the identity with the initial state remains very high (r > 0.95), consistently with the character of (quasi)-stable state of both RPM and On Ground samples.

These results strongly suggest that all the experimental samples are (still) proper MCF7 cell populations, even after the observed morphological/functional transition had occurred in the simulated microgravity field, leading to different morphological phenotypes. The invariance of these quasi-stable states is confirmed by the strong mutual correlations among cell phenotypes (OG, RPMAD and RPMCLUM) observed at 12 different time points (OG1h, OG2h, OG6h, OG24h; RPMAD1h, RPMAD2h, RPMAD6h, RPMAD24h; RPMCLUM1h, RPMCLUM2h, RPMCLUM6h, RPMCLUM24h) we considered (Table 2). Looking at the autocorrelation time course of each cell population, as expected in the case of quasi-stable states, we observe a modest but statistically significant time drift from the starting condition. Figure 5 reports these drifts in terms of logarithm of time (abscissa) and correlation values with initial time (ordinate).

The time drift for all the three conditions is quite moderate, albeit highly significant (*p* < 0,001). We found that, in respect to the initial condition (OG), the adherent population at 24h (RPMAD24) showed the lowest correlation. However, correlation coefficients were in any case close to the unity (r ≥ 0.950). This result confirms that, even after 24h of simulated microgravity-exposure, cell populations display a physiological quasi-stable state, notwithstanding the dramatic changes in shape and function they display. As it is evident in Fig. 5, all conditions (OG, RPMAD and RPMCLUM) show very similar time drifts, thus indicating their comparable stability.

**Fig. 6:**
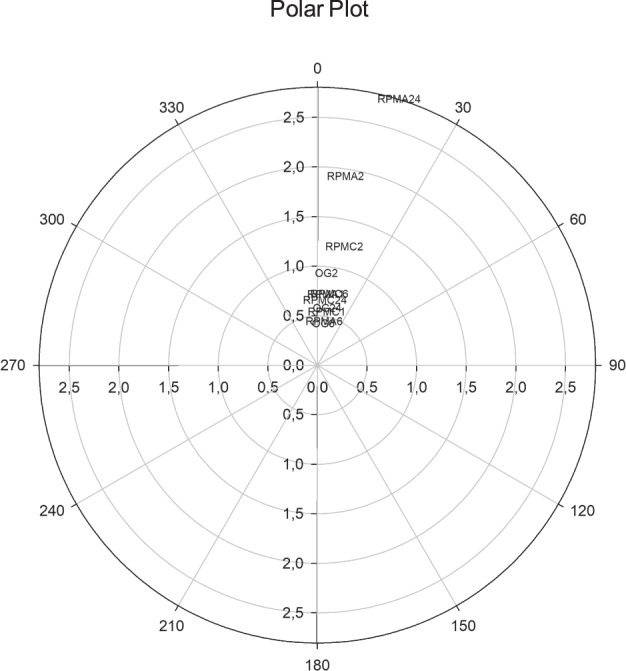
Euclidean and Angular distances in gene-expression patterns. The plot reports the Euclidean distance (see also Supplementary Fig. 3) from the centre computed over the gene-expression values (concentric circles) and the angle of deviation (the angle between OG1 and different profiles having Pearson r with OG1 as cosine) from baseline (OG1) condition. Data are computed at different times for both RMPCLUM and RPMAD (1, 2, 6, 24 h). The overlapping or partially overlapping experimental points are the following: OG6, OG24, RPMA1, RPMA6, RPMC1, RPMC6, RPMC24.

The projection of the gene-expression profile on a Polar Plot centred on OG baseline condition (Fig. 6), allows a synthetic global view of the transition across different states (RPMCLUM and RPMAD), at diverse time points (1, 2, 6, 24 h). This is made possible by the unique property of Polar Plots of reporting simultaneously profile (the ordinate is the angle with OG) and quantitative (the abscissa is the Euclidean distance from centre) information. The coordinated departure from the centre is the signature of the divergence from the basic OG condition and actually ‘measures’ how relevant the microgravity-induced rewiring is in gene-expression pattern. Deviations from the profile identity (angle = 0°) are only slight, and the most ‘deviating’ sample (RPMAD24, angle = 17°), shows also the higher Euclidean distance from the centre. Therefore, the phenotypes emerging in simulated microgravity are not due to contingent, stochastic changes, given that they correspond to different sub-attractors in gene-expression space, as confirmed by the statistically significant correlation between Angle and Euclidean Distance (r = 0.682, *p* < 0.02). That finding further outlines the coordinated motion involving the relative proportion of single gene-expression values, as reported further on.

#### Perturbation experiments show that critical transition leading to distinct physiological states proceeds according to Hysteresis

**Fig. 7:**
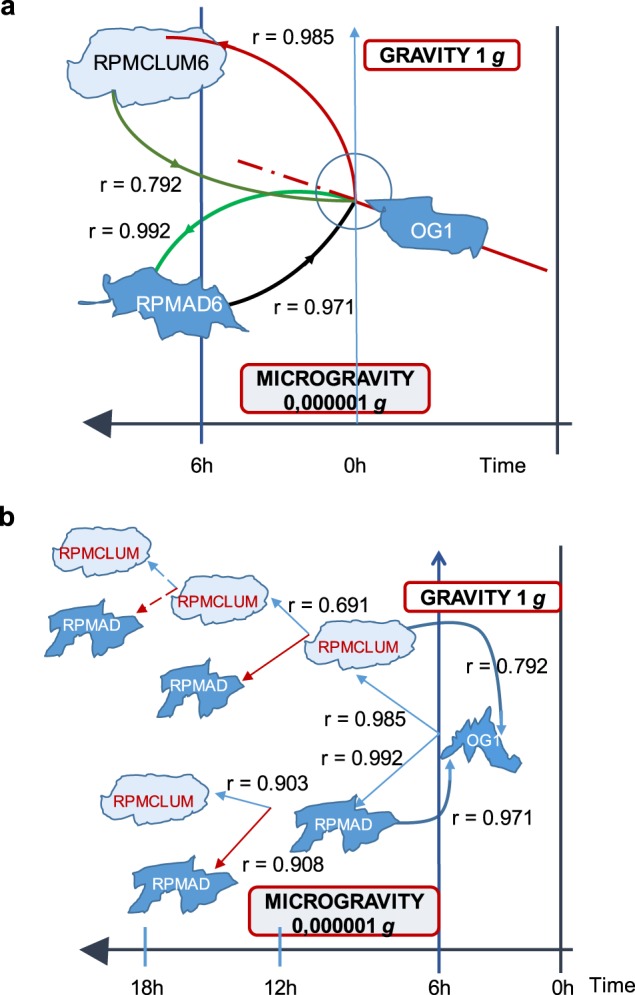
Phenotypic transition across different gravity fields. In panel **a**, the phenotypic transition occurring in microgravity is schematically represented. Pearson correlation coefficients are reported for gravity to microgravity transition, as well as for the reverse path, during which both RPMAD and RPMCLUM recover their native morphology. The recorded differences suggest that cells during the recovery process follow a different path (hysteresis). In panel **b**, cells of each population further split into two phenotypes when separately located again in microgravity: an adherent and a clump one. As observed during the first transition (from OG to both RMPCLUM and RPMAD), Pearson coefficients still preserve a highly correlated profile when compared to OG values.

Cells in simulated microgravity are partitioned into two different phenotypes. Transitions from OG to RPMAD and RPMCLUM are associated, after 6 h of permanence in simulated microgravity, with correlation coefficients of 0.985 and 0.992, respectively. In the reverse path, cell clusters conditioned in simulated microgravity field acquired de novo their native morphological phenotype when reinstated on normal gravity. Figure 7a shows schematically this process, and the related coefficient correlation values.

The RPMAD sample was the most correlated with OG1 when replaced in normal gravity (after 6 h on ground, correlation with OG1, r = 0.971). On the contrary, the recovery experiment relative to clumps population (correlation with OG1, r = 0.792), shows the lowest correlation value. This means clumps cells most likely correspond to a physiological state ‘more distant’ from baseline (OG).

According to Pearson’s correlation coefficients, these findings suggest that the ‘coming back’ process follows a different ‘path’ with respect to the initial move-away from the baseline condition, thus supporting the hysteresis-like behaviour underlying the reversion process. This means that each physiological state retains a ‘memory’ (i.e. a persistence of the acquired new phenotypic state) of the travelled trajectories.

In the subsequent experiment, when each cell population, already obtained in simulated microgravity, is relocated again into the simulated microgravity field, cells of each population further split into two phenotypes: an adherent and a clump one (Fig. 7b). The same process has been sequentially repeated many times, and in each case, we always observed a superimposable phenomenon: a single cell phenotype gives origin to two different morphological clusters. As observed during the first transition (from OG to both RMPCLUM and RPMAD) previously discussed, Pearson coefficients show small fluctuations from the unit value.

**Fig. 8:**
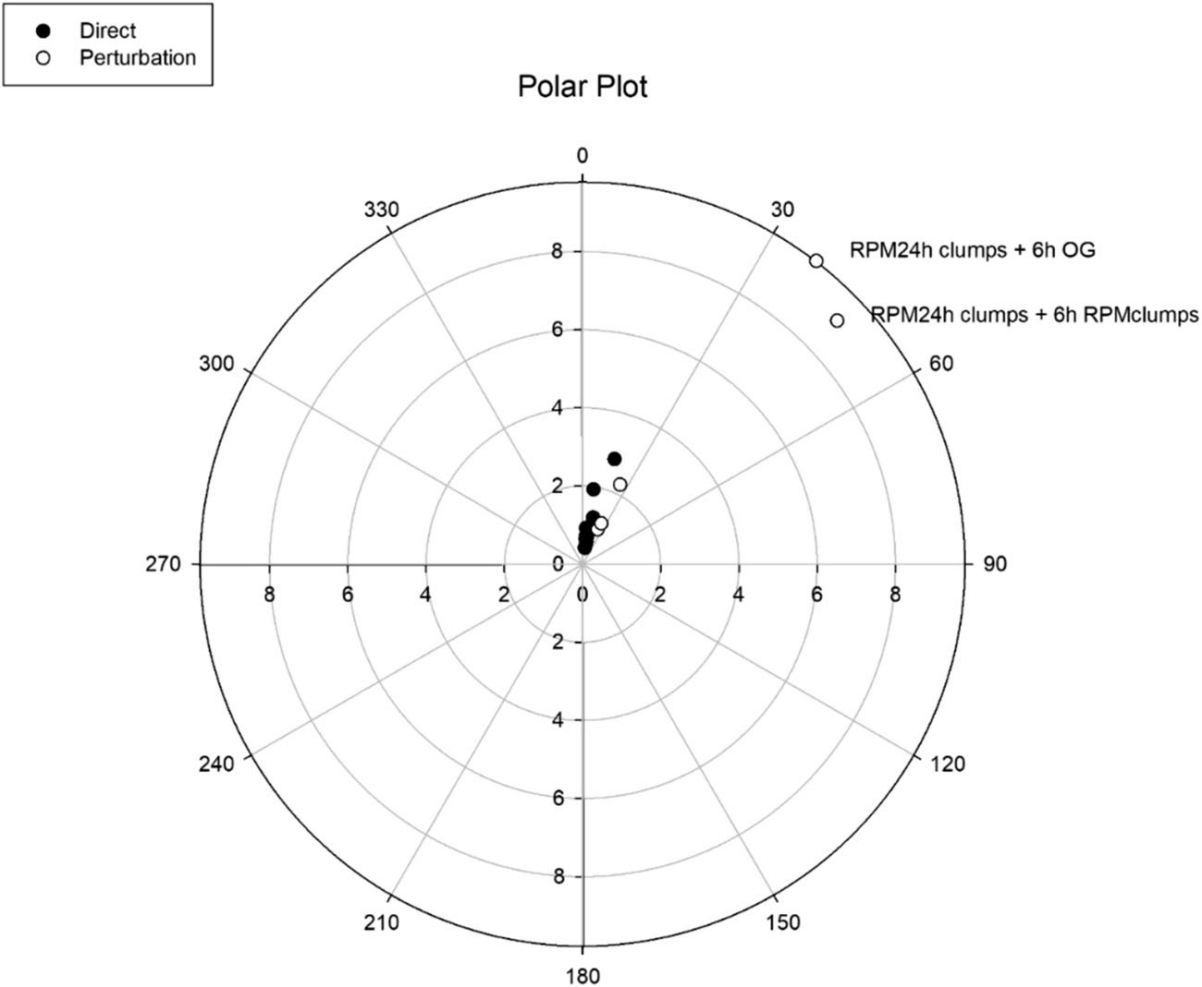
Calculated Angle and Euclidean distances in MCF7 gene-expression values during first (from gravity to microgravity, direct group, black marble) and secondary (from microgravity to gravity or from microgravity to a further period of microgravity conditioning, perturbed group, white marble). The figure reports the Angle and Euclidean distance with OG1 (baseline) relative to both Direct and Perturbed population. Direct samples include both RPMAD and RPMCLUM after 1, 2, 6, 24 h in microgravity and control OG at 1 h. Perturbed cells includes the two phenotypes, RPMAD and RPMCLUM—conditioned in microgravity for 24 h—that were forced to regain (1) their native 1 g condition (evaluated after 6 h of 1 g conditioning); or, (2) to be again seeded in microgravity for 6 more hours, after being partitioned as single phenotype. The plot reports the Euclidean distance from the centre computed over the gene-expression values (concentric circles) and the angle of deviation (the angle between OG1 and different profiles having Pearson r with OG1 as cosine) from baseline (OG1) condition. The two most extreme (for both Euclidean Distance and Angle) displacements from baseline are relative to ‘Clumps-Organoid’ cells that are confirmed to be the phenotype most diverse from baseline in both morphology and gene-expression spaces.

For all the analysed conditions, the strict relation between Angle and Euclidean distance from OG1 was established for cell samples moving away from their respective equilibrium condition: (a) ‘direct’ group (group A), in which cells move from normal gravity to simulated microgravity; observations were performed after different times (from 1 to 24 h) spent in simulated microgravity (Fig. 8, black marbles). (b) ‘perturbed’ group (group B), which includes the two phenotypes- RPMAD and RPMCLUM, conditioned in simulated microgravity for 24 h—that were forced to regain (1) their native 1 g condition (evaluated after 6 hours of 1 g conditioning); or, alternatively, (2) to be again seeded in simulated microgravity for 6 more hours, after being partitioned as single phenotype (Fig. 8, white marbles). In the Fig. 10, results are projected on a polar plot having as abscissa the Angle distance with OG1, and in ordinate the Euclidean distance from the same baseline condition. In the direct group, the departure from the equilibrium is associated with minimal changes in both Angle and Euclidean distance, suggesting that this transition induce only a minor rewiring of the gene-expression pattern. Cells in the perturbed group show greater Angle and Euclidean distance values, with respect to the direct group.

## Discussion

Several reports have shown that cells are sensitive to a gravity field and respond to microgravity by displaying changes entailing many phenotypic traits, including morphology, biochemical pathways and gene expression. Investigations so far performed aimed at ascertaining if the gravity field affects specific, selected clusters of genes, tightly linked to critical pathways. Several genes have been found to be deregulated, even if it is quite difficult to recognize the function they could actually play in that condition, as the overall set of ‘deregulated’ genes is quite heterogeneous.^24^ Moreover, studies searching for genes specifically involved in critical pathways usually affected by microgravity (like bone resorption processes) found only a loosely correlation with the investigated phenomenon, while discovering several ‘altered’ genes not involved in the specific physiological aspect under study. Furthermore, a modest overlapping has been noticed among findings obtained by different reports: only few genes were reproducibly reported to be up or down regulated when cells are exposed to microgravity.^25^

However, a few studies have shown that in microgravity the gene-expression pattern undergoes wide modifications, entailing thousands of genes and several unrelated pathways,^26^ so far evidencing that the gravitational field can interfere with the whole system. Therefore, it is quite unlikely that a reductionist approach, i.e. the search for a few key genes whose expression could explain the modifications so far observed in microgravity, could provide an affordable ‘explication’ of what really happens in weightlessness condition. Gene-expression changes are probably secondary to disturbances in the cell-microenvironment cross talk. Indeed, the gene-expression pattern has been observed to be severely altered also as a consequence of cell transfer into a new culture environment, though it consisted of approved biologically inert and sterile material, and although the cells had up to several hours to adapt to the new conditioned medium.^27^ These findings suggest that mechanical interactions between cells and their microenvironment play a pivotal role in shaping gene activity.^28^ In fact, the gravity-related effect on living cells and tissues is likely a systemic one because it is mediated by early changes involving the cytoskeleton architecture, a complex and dynamic structure not only able in sensing any disruption occurring in the mechano-balance between cells and their microenvironment,^29^ but also highly sensitive to modifications in the non-equilibrium dynamics. Indeed, cytoskeleton changes reflect universal non-equilibrium dynamics of living cells,^30^ as it undergoes very early (already after only 6 min)^31^ - self-organized arrangements in response to microgravity,^32–34^ and such changes are likely to be transmitted to the nucleoskeleton, thus influencing chromatin structure and gene activity.^35,36^ Overall, these data hint that alterations of nuclear structure-function interrelationships can occur because of microgravity-mediated perturbations.^37^

Results reported herein demonstrate that:

- As previously reported for endothelial,^38^ thyroid^39^ and bone cells,^40^ breast cancer MCF7 cells exposed to simulated weightlessness undergo significant morphologic and phenotypic changes. Indeed, cells were equally (50% each) partitioned into two quasi-stable populations, displaying different morphological features, as confirmed by quantitative morphological analysis and CSK remodelling. Moreover, adherent and clumps cells showed a significantly different behaviour when proliferation and apoptotic rates were kept in consideration, as previously observed in other breast cancer models.^6^ Namely, adherent cells showed no difference in apoptosis rate when compared to OG samples, while a significant increase in programmed cell death has been observed in RPMCLUM cells. Yet, this apoptotic trend is partially compensated by sustained proliferation, as RPMCLUM are still viable and proliferating.
- Overall, those modifications look like transient, adaptive changes as cells promptly recover their native morphological phenotype when replaced in normal gravity. System analysis of gene expression during the transition from one state to the other shows only a slight decrease in Pearson correlation values, which were always >0.95 for all samples considered. This finding stands for the robustness of the MCF7 genome expression pattern across different gravity conditions, thus confirming the self-organized behaviour of complex living systems, which exhibit super-, near- and sub-critical states corresponding to the ensemble of high-, intermediate-, and low-variance gene expression, respectively, and their coherent oscillatory dynamics.^41^ The recent paper by Gershovic et al.^34^ confirms our findings: while F-actin cytoskeleton is dramatically remodelled under microgravity in human bone marrow cells, the normal gene-expression profile is still preserved. This implies that genome activity displays a relevant resilience in respect to environmental changes.
- In principle, the hysteresis-like process performed by these transitions would hint for a critical transition, remnant of the first order transitions observed in chemical/physical systems, given that hysteresis-driven systems are predicted to show abrupt state transitions over time.^42^ However, our findings demonstrated that such critical transition does not involve the gene-expression pattern. Therefore, it is likely that microgravity-induced transformations will take place at the epigenetic/post-translational level, involving the cytoskeleton as well as a few pathways, which are likely shaped by non-equilibrium dynamics.
- Overall, that finding stands for the existence of physical-dependent alternative stable states, a feature exhibited by complex systems when one or more of its state variables responds to environmental change by a backwards folding curve, displaying bistability and hysteresis.^43^ The existence of alternative stable state in cellular physiology is a further evidence of the complex relationships between genotype and phenotype, as stressed by several studies.^44–46^
- A hysteresis pattern indicates true alternative attractors if the response of the system is fast enough relative to the rate of change in the control factor. This is precisely the case, as CSK remodelling happens even after 6 min^32^ of microgravity exposure, while pathways and gene-expression modifications have been recorded since after the first hour of microgravity conditioning.^47^ An in depth assessment of the alternative stable states enacted by removing the gravity constraint will require further studies. Namely, conclusive experimental approaches should investigate: (1) the existence of different parameter thresholds for back- and forward shifts (test for discontinuity), (2) state transitions after perturbations (test for non-recovery), and (3) sensitivity of the stable end state to initial conditions (test for divergence).
- In absence of gravity the cytoskeleton cannot find a proper equilibrium stabilization, losing its native orientation, as previously highlighted.^47^ It is likely that cells can ‘sense’ changes in the gravity field through early modifications in the non-equilibrium dynamics that govern the CSK architecture. In turn, changes in CSK structures can transduce the physical perturbation through the mechanosensory cellular system,^48^ thus influencing many cellular processes and even the gene-expression profile. As expected, we observed dramatic changes in CSK architecture after exposition to simulated microgravity. Moreover, such changes significantly diverge between the two clusters in which the native MCF7 sample splits after simulated microgravity exposure. In principle, such differences can explain why the two cell types display bistability, i.e. different phenotypic and morphological features, given that CSK changes are associated with post-translational and/or epigenetic modifications. Indeed, we and others,^6,7,9,34^ observed that the two cell phenotypes show differentially regulated pathways. Changes in CSK architecture are instrumental in shaping different cell morphologies, and, in addition, it is conceivable that such shape modifications may in turn influence cell growth and metabolism as well as gene expression.^49,50^ Given that CSK remodelling in microgravity is an everlasting phenomenon (due to non-equilibrium dynamics),^51^ this would lead to an endless rearrangement of the gene-expression pattern, while cells will be oscillating in between different morphological configurations. Therefore, shape and functional traits arise freely as a consequence of the non-equilibrium that govern the dynamics of self-organizing living structure in absence of gravity.^52^
- Our findings highlight the relevance of physical constraints in driving cell-fate commitment. In absence of such constraints, the system is unable to choose between two different phenotypes, thus leading cells to be segregated into different clusters. In other words, the genotype does not determine by itself the phenotype but requires additional, environmental cues to proper finalize cell differentiation.^53^ In fact, the absence of (physical) constraint impairs proper differentiation and eventually enacts opposite effects on different cell clusters. For instance, we show that apoptosis is almost inhibited in adherent cells while increasing in floating (clump) cells, as previously observed.^54^ Likewise, previous reports have highlighted that other cell types may undergo both differentiation or apoptosis.^55^ In absence of gravity, the correct developmental/differentiation pathways leading to an organized individual are therefore severely disturbed and hindered.^56^ These results cast on doubt that normal embryological and developmental life processes could take place in absence of Earth gravity.
- Our major finding is that the morphological and functional changes go together with a coordinated motion in the gene-expression phase-space, without entailing major changes in gene activity. The trajectories of the different conditions in the gene-expression phase space are consistent with the existence of sub-attractors within the main attractor correspondent to the cell type we investigated (MCF7 cells). These results suggest that manipulation of the environmental field can result in deep behavioural and phenotypic changes, while preserving the overall coherence (i.e., invariance) of the gene-expression pattern.

In summary, earlier studies have pointed out the hypothesis that under different microenvironmental conditions—in which cells are exposed to a different regimen of tensional forces—both morphology and biological properties of cells are reversible. Namely, Bissell and colleagues^57^ showed that the malignant phenotype of a breast cancer cell line was reversible based on modulation of signaling from the microenvironment. It is worth noting that such pioneering study demonstrated that the rewiring of the cancerous phenotype occurred without involving significant changes in genome activity. These findings have been then recognized by other studies.^58^ Herein, we also demonstrated that changes in the tensional homeostasis of a cell population—mostly driven by changes in the gravitational field—can also lead to a successful reversion of their native phenotype.

Studies performed in simulated microgravity can likely provide useful insights both for grasping biomedical processes occurring in space and for widening the knowledge of basic physical principles governing the organization of biological systems. Namely, cell-fate specification may undergo a switch from a bifurcation point (where the system is deprived of the gravity constraint), leading hence to morphological and functional different phenotypes. It is worth noting that such dramatic change can occur without entailing the overall genome coherence, i.e. without involving a significant rewiring of the gene-expression pattern. The obvious corollary of such finding is that you do not require any significant gene activity change for achieving an even outstanding phenotypic modification. Indeed, different phenotypes of MCF7 cells exposed to simulated microgravity still retain their “genomic identity”, as pinpointed by the invariance in their Pearson’s coefficient values. Instead, even “opposite” (adherents versus floating) phenotypes can be shaped by physical constraints only by acting at a post-translational level. It is arguable that an instrumental role in such changes is mostly supported by early changes in CSK architecture. Indeed, remodelling of CSK is tightly linked to the cell biochemical machinery and is highly sensitive to any changes of the non-equilibrium dynamics governing the behaviour of the living system. These findings can help us in understanding the functional and morphological modifications occurring when some physical, tissue constraints (stiffness, viscosity, etc.) are deregulated and consequently cells lose their constraints-dependent properties, as observed in cancer.^28,59^ Indeed, in biology a regime shift, i.e. the occurrence of bistability and hysteresis, is usually attributed to an intricate mix of internal processes and external factors/forces, closely interwoven and tough to untangle.^60^ Yet, studies performed on microgravity provide an elegant model that allows investigating in isolation the role of a physical constraint (the gravity field) during cell phenotypic transitions, avoiding other confounding factors, namely the intrinsic biochemical/genetic activity of cells.

## Methods

### Simulated microgravity condition through Random Positioning Machine (RPM)

Microgravity conditions were simulated by a Desktop RPM, a particular kind of 3D clinostat,^61^ manufactured by Dutch Space (Leiden, The Netherlands). The degree of microgravity simulation depends on angular speed and on the inclination of the disk. These tools do not actually eliminate the gravity but allow you to apply a stimulus rather than a unidirectional omnidirectional 1 g. Effects generated by the RPM are comparable to those of the real microgravity, provided that the direction changes are faster than the response time of the system to gravity field. The desktop RPM was located in a standard incubator (to maintain temperature, CO_2_, and humidity levels) and connected to the control console.

### Cell culture

MCF7 human breast cancer cell line was purchased from European Collection of Cell Cultures (ECACC, Sigma–Aldrich, St Louis, MO, USA). Cells were seeded into Nunc^®^ OptiCell™ Cell Culture Systems, gas-permeable cell culture disks (Thermo Scientific, Rochester, USA). MCF7 were cultured in Dulbecco’s modified Eagle’s medium (DMEM, Euroclone Ltd., Cramlington, UK) supplemented with 10% Fetal Bovine Serum (FBS, HyClone Laboratories, Logan, UT, USA), 200 mM L-glutamine, 100 IU/ml Penicillin and 100 μg/ml Streptomycin (all from Euroclone Ltd., Cramlington, UK). Cells were allowed to incubate for 24 h and then, cell culture disks were fixed onto the RPM, as close as possible to the center of the platform, which was rotated at a speed of 60°/s using the random mode of the machine. On ground control (1 g static cultures) and RPM cultures were kept in the same humidified incubator at 37 °C in an atmosphere of 5% CO2 in air. The experiments were performed for 1, 2, 6 and 24 h. After simulated microgravity exposure, cell clumps swimming in culture supernatants were found, in addition of adherent cells, separately collected and characterized. Moreover, cell clumps were partially reseeded into a normal gravitational field and partially relocated in simulated microgravity conditions. These experiments were performed for 6 and 24 h.

### Proliferation and cell cycle

For proliferation assay, cells were seeded in 35 mm Petri dish at a concentration of 5 × 10^4^ cells/dish in a complete medium. Cells cultured on ground or on RPM for 24, 48 and 72 h were counted with a particle count and size analyzer (Beckman-Coulter, Inc., Fullerton, CA, USA) and by a Thoma hemocytometer. Three independent experiments in duplicate were performed. For cell cycle analysis, cell clumps were collected and centrifuged and pellets were trypsinized and washed twice with PBS (Phosphate Buffered Saline, Sigma–Aldrich, St. Louis, MO, USA). Adherent and ground control cells were trypsinized and washed twice with PBS. Cells were fixed with 70% ethanol at 4 °C for 24 h and stained with DNA PREP Stain (Beckman Coulter, Fullerton, USA) at 4 °C overnight. Stained cells were measured by flow cytometry. Cell cycle analysis was performed three times.

### Optical microscopy

Cells cultured on ground or on RPM, were photographed with Nikon Coolpix 995 digital camera coupled with Zeiss Axiovert optical microscope. The images were obtained with a ×320 magnification and saved as TIFF files. Cell clumps were collected, washed in PBS and deposited onto a clearly-defined area of a glass slide using a Shandon CytoSpin™ 4 Cytocentrifuge - Thermo Scientific, while maintaining cellular integrity. Cell clumps, adherent and on ground control cells were fixed in 4% paraformaldehyde for 10 min at 4 °C, photographed, as previously described, and used for image analysis.

### Image and fractal analysis

Image analysis was performed on 10 images for each group of cells. In each image, single randomly chosen cells (50 for each group) were contoured with a fine black marker by different researchers, simply scanned and catalogued according to the time of study: 24 and 72 h. Thus, we decided to perform a semi-automatic analysis, coupling the expertise of researchers with a computerized parameterization method. All the images were processed by Adobe Photoshop CS4. All the pictures (i.e. all the sheets of the groups, for each time point) were resized at 2560 × 1920 pixels according to original scale of image acquisition. For each black contoured cell, edges were refined. Then cells were black filled and threshold was adjusted in order to exclude from the image other cells and background. For each time point a single sheet of all the cells considered was created. To obtain single cell shape parameters (area A, roundness, solidity, fractal dimension FD), ImageJ v1.47 h software was used. Then, the software analyzed single cells, by the function “shape descriptor.” In addition to area A, we calculated roundness (Eq. 1) and solidity (Eq. 2):

1 $${\mathrm{Roundness}} = \frac{{4A}}{{\pi \sqrt {ma} }}$$

2 $${\mathrm{Solidity}} = \frac{A}{{CA}}$$

Where A is the area of the cell, ma is the major axis, and CA is the convex area, namely the area of the convex hull of the region. The convex hull of a region is the smallest region that satisfies two conditions: (a) it is convex (b) it contains the original region.

As for FD, it was obtained by means of box counting method using FracLac plugin (Eq. 3):

3 $$FD = \mathop {{\lim }}\limits_{\varepsilon \to 0} \left[ {1 - \frac{{\log [L_\varepsilon (C)]}}{{\log \varepsilon }}} \right]$$

where C is the considered curve, L is the length of the curve C, and ε is the length of the segment used as unit to calculate L.

Single graphs about roundness, solidity and FD were obtained for each set of images.

### Immunofluorescence

To describe the organization of cytoskeleton proteins and adhesion molecules in OG, RPMAD and RPMCLUM MCF7 cultured cells, we performed immunofluorescence experiments using primary antibody against β1 integrin, cofilin, tubulin and vinculin. Cell nuclei were stained with TO-PRO-3 (TO-PRO3 iodide fluorescent dye 642/661 (1:5000 in PBS, Invitrogen, cat. T3605, Carlsbad, CA, USA), and F-actin was visualized using Rhodamine Phalloidin (Invitrogen Molecular Probes Eugene, 1: 40 dilution). Briefly, cells were fixed with 4% paraformaldehyde for 10 min at 4 °C, and washed twice for 10 min with PBS. Cells were permeabilized for 30 min using PBS, 3% BSA, 0.1% Triton X-100, followed by anti-vinculin (7F9): sc-73614 (Santa Cruz Biotechnology) 1:200; anti-β1 integrin, (M106) sc-8978 Santa Cruz Biotechnology) 1:200; anti-cofilin (FL-166) sc-3377; Santa Cruz Biotechnology) 1:200; anti-tubulin (Sigma T5168) 1:1000, staining in PBS, 3% BSA at 4 °C overnight. The cells were then washed with PBS, and incubated for 1 h at room temperature with appropriate secondary antibody FITC or TRITC conjugated (Invitrogen Molecular Probes Eugene, Oregon). Negative controls were processed in the same conditions besides primary antibody staining. Cells were then washed in PBS and mounted in buffered glycerol (0.1 M, pH 9.5). Cells stained with anti-tubulin antibody were analyzed using a Zeiss Fluorescent Microscope. The images were scanned under 40x objective.

### Confocal microscopy analysis

The distribution pattern of F-actin, β1 integrin, cofilin, and vinculin has been analyzed by confocal microscopy. The analysis was conducted using a Leica confocal microscope TCS SP2 (Leica Microsystems Heidelberg GmbH, Mannheim, Germany) equipped with Ar/ArKr and He/Ne lasers. Laser line were at 543 nm and 488 and 633 nm for TRITC, FITC and TOPRO iodide −3 excitation, respectively. The images were scanned under ×20 or ×40 oil objectives. To analyse the co-localization of F-actin and vinculin colour channels were merged with the Leica confocal software.

#### RNA extraction and gene-expression analysis

Total RNA was isolated from MCF7 cells using Trireagent (Ambion, Thermo Fisher Scientific, Carlsbad CA) and one microgram of RNA was reverse-transcribed using the High-capacity cDNA Reverse-Transcription Kit (Thermo Fisher Scientific, Carlsbad CA). cDNA was used for quantitative RT- PCR (qRT-PCR) analysis using ViiA 7 Real-Time PCR System (Thermo Fisher Scientific) and SensiFAST Probe Lo-ROX (Bioline). Each amplification was performed in triplicate and the average cycle threshold (Ct) was used for analyses. Taqman assays (Thermo Fisher Scientific), chosen with the criterion of best coverage, were used. Genes analyzed and Assay IDs are listed in Supplementary Table 2.

### Apoptosis

Cell clumps were collected, centrifuged and pellets were trypsinized and washed twice with PBS. Adherent cells and ground control cells were trypsinized and washed twice with PBS. The cells were stained with FITC labeled annexin V/7-AAD (7 aminoactinomycine-D) according to the manufacturer’s instructions (annexin V/7-AAD kit; Beckman CoulterTM, Marseille, France). Briefly, a washed cell pellet (5 × 10^4^ cells/ml) was resuspended in 500 μL binding buffer; 10 μL of annexin V together with 20 μL 7-AAD were added to 470 μL cell suspension. The cells were incubated for 15 min on ice in the dark. The samples were analyzed by flow cytometry. Apoptosis assay was performed three times.

### Statistical analysis and mathematical modelling

All experiments were performed in triplicate. Data were expressed as mean ± standard error (SE) and as mean ± standard deviation (SD). Data were statistically analyzed with the Student’s *t*-test and ANOVA test followed by the Bonferroni post-test for multigroup comparison, when appropriate. Differences were considered significant at the level of *p* < 0.05. Statistical analysis was performed by using GraphPad Instat software (GraphPad Software, Inc.; San Diego, CA, USA).

### Reporting summary

Further information on research design is available in the Nature Research Reporting Summary linked to this article.

## Supplementary information

Reporting Summary Checklist

Supplementary informations

## Data Availability

All relevant data are within the paper and its Supporting Information
