# Supplementary material for: Phenotypic transitions enacted by simulated microgravity do not alter coherence in gene transcription profile
Source: NPJ Microgravity. 2019 Nov 21;5:27. doi: 10.1038/s41526-019-0088-x (PMC6872750; doi:10.1038/s41526-019-0088-x)
Supplement: Supplementary file 2 — Supplementary informations [file 41526_2019_88_MOESM2_ESM.pdf]

Supporting information

Supplementary Methods

The experimental model. We used a coarse-grain statistical metrics for estimating the behavior of a gene ensemble, adopting a statistical mechanics-inspired model of biological regulation (1). Accordingly, whole genome expression is ruled by self-organization processes, as described by the Self Organized Criticality (SOC) theory (2).

Briefly, SOC considers a cell-fate decision-making model where diverse cell-fate options are first generated by sorting out of various transcriptional programs, and then a cell-fate gene module is selectively amplified when the network system approaches a critical state.

It is generally assumed that each differentiated state – a cell phenotype - corresponds to a very stable gene expression pattern. This condition implies that the system displays *high coherence values*, recognizable by *very strong correlation between key parameters* (transcriptome profiles, among others) obtained from independent samples belonging to the same tissue (3). Supplementary Methods figure 1 refers to MCF7 cell line and clarifies this point: the axes refer to two independent MCF7 samples whose single gene expression values are the points of the graph (around 23000, expression values in logarithm units), the d-value corresponds to the range (box size) of variation, inside which the correlation

(Pearson coefficient, r) is computed (3). The correlation computed overall is near to unity (r = 0.98), and declines at decreasing range of variation. The inset on top left angle of the figure shows the reaching of a plateau correlation at d = 0.45. This remark outlines how correlation values are tightly dependent on the observation scale, and it is consequently mandatory to choose a proper level of observation in setting the experimental investigation. The scale dependence of the correlation is instrumental to keep alive both the functionality of the tissue (the specialized physiological function asks for an invariant "ideal" pattern of gene expression) and the flexibility required to adapt to changing microenvironment, tuning the specific gene expressions at the small scale. This fine-tuning does not alter the global profile invariance and corresponds to the scattering of the points around the identity line reported in Supplementary Methods figure 1. Even if (slightly) less precise, this correlation holds even at the single cell scale (4), and changes in single cell correlation structure are instrumental to predict cell fate transitions (5).The dispersion across the identity line corresponds to the equilibrium around a definite physiological state and keeps invariant the correlation coefficient between two snapshots of the cell population. Only when the system undergoes a transition toward another state, with a concomitant wide modification in gene expression pattern, this correlation changes in a relevant manner. In principle, any directed modification of the phenotypic state is sustained by the coordinated change of activities at a large number of gene loci across the genome, altering the relative proportion of gene expression, thus affecting their mutual correlation. Because

genes influence one another's expression by means of a network of regulatory interactions, genes cannot alter their expression in an *independent* manner, and transcriptomes (which are the measurable *proxies* for genome-wide gene activation profiles and hence, for cell states) can change only in *a highly constrained manner* (6).

Supplementary Methods figure 1

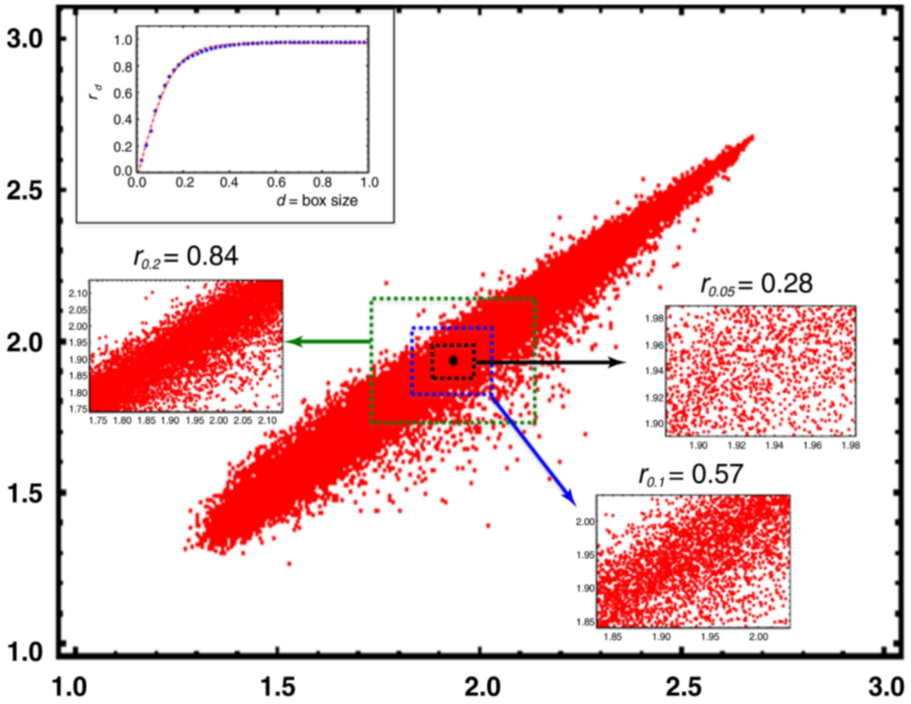

Supplementary Methods Fig. 1. Pearson's correlation between independent samples of the same cell type. The figure reports the correlation between two independent samples of MCF7 (axes of the plot) in terms of expression levels of around 23000 genes (vector points). The overall correlation is near to unity (r = 0.98) consistently with the existence of a main attractor correspondent to the cell-kind. At smaller scale of variation (different values of d, box-size) the correlation decreases,

given the local expression fluctuations needed to cope with slight environmental changes (genetic noise) obscure the existence of an 'ideal expression profile'. The top-left inset shows how the attractor structure is fully present at d = 0.45 (modified from (11)).

It is crucially important to keep in mind the hierarchical character of the definition of 'state': each cell type can be defined, in dynamical terms, as an 'attractor', i.e. a minimum energy configuration to which the system returns when the effect of a perturbation (e.g., a drug, a physical cue) fades away. We can imagine this attractor as a deep valley of a rugged energy landscape (6). The minimum-energy bottom of this valley, in analogy with protein three-dimensional configuration, is not a single point but admits several sub-attractors that can be considered quasi-stable states in which the system might reside (7). The presence of multiple, quasi-identical solutions of energy minima at the bottom of the energy landscape is instrumental to guarantee adaptation to environmental changes. In principle, a physical constraint can modify the phenotypic state, without modifying the cell type. This would correspond to a (relatively small) change in between gene expression profiles correlation coefficient (hypothesis b).

We set us in investigating such changes in correlation values between gene expression profiles to check for a 'gene expression profile counterpart' of the morphological changes induced by microgravity. In order to have a magnitude estimation of such changes, we are describing herein previously published correlation values between transcriptomes of the same tissue in two physiological

states (Supplementary Methods figure 2a), together with the correlation between two different tissues relative to the same animal (Supplementary Methods figure 2b). Passing from the same tissue in different physiological states (jumping between two sub-attractors of the same main tissue attractor) to two different tissues (two different main attractors), the correlation coefficient drops from 0.97 to 0.30. The near to unity correlation of different physiological states relative to the same tissue (Supplementary Methods figure 2a), depends from the existence of a tissue-specific 'ideal' profile shared by the different states, which disappears in the bottom panel representation (Supplementary Methods figure 2b) (8).

Supplementary Methods figure 2

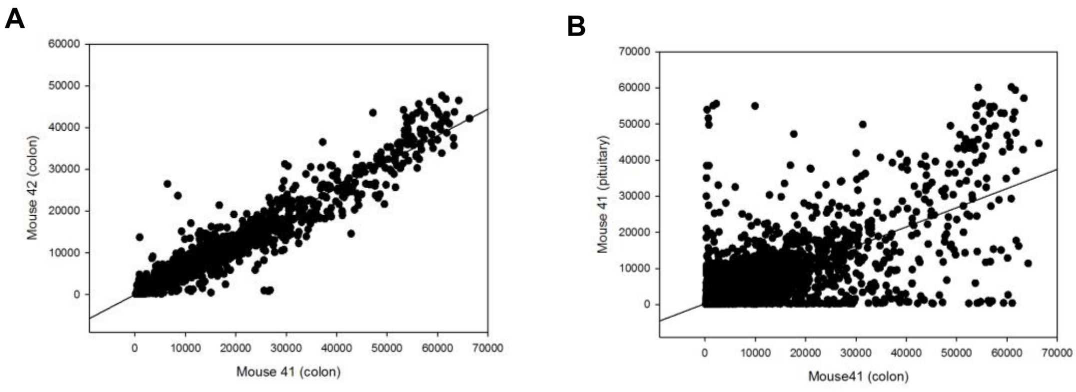

Supplementary Methods Figure 2. Gene expression patterns and Pearson's correlation coefficients. Fig. 2a reports the gene expression profiles of two independent (obtained from different animals) samples of the same tissue (colon). Fig.2b depicts on x-axis the gene expression profiles relative to two different tissues (colon and pituitary gland) pertinent to the same animal. In the top-

panel, correlation is near to unity (r=0.97), whereas it drops to r=0.30, when different tissue of the same animal are considered (bottom panel) (modified from (8)).

In physical-mathematical terms, we consider every stable transcriptome as an 'attractor', to which the system tends to come back when perturbed. In biological terms, we can define an 'attractor' as a stable, observable cell phenotype in which all gene regulatory interactions show slight oscillations, preserving the overall coherence. Accordingly, the switch from one stable cell state to another corresponds to a 'transition' from one attractor to another (9). The invariance of gene expression profile in respect to perturbations (resilience) comes from the existence of robust gene interaction network. This implies that a tissue observed at different time intervals keeps a near to unity autocorrelation in respect to its initial (t0) profile, displaying only minor variations, mostly due to stochastic fluctuations. We must outline that the autocorrelation is detectable given we select a *sufficiently wide range of mean expressions* of any choice of probe genes, instead of analysing the overall transcriptome landscape, as already reported in greater details (2, 10, 11). Therefore, here we focused on an appropriate set of 26 gene expression data. These 26 genes – independently from the biological function they fulfil - were chosen with a sufficiently diverse average expression range of variation (d = 0.60 in logarithmic units, well inside the plateau correlation range, see inset of Supplementary Methods figure 1) to highpoint the attractor-like behaviour. In the full-rank situation each sample is thus a 26-component, real-valued vector. It is

worth noting to recall that cell cultures *in vitro* do not perfectly adhere to the ideal portrait. They are more aptly defined as 'quasi' equilibrium states, as we do expect a slow decay of autocorrelation in time reflecting the impossibility to maintain forever viable an *in vitro* cell culture (11). On the other hand, a lethal disruption of the gene interaction networks is expected to destroy the between profiles correlation. We selected genes relative to a coherent network that, as expected, gave rise to very high average between genes correlation (r = 0.834, SD = 0.07). This between genes strict correlation is instrumental for dealing with eventual missing values. Each pairwise comparison between profiles can be only computed over the components (gene expression data) esteemed in both samples. The mutual correlation between gene expression values, reassures us that the obtained results are largely invariant for small variations of the considered genes due to eventual missing values. According to the aforementioned approach, we planned to investigate the following points:

1. Assessment of quasi-stability of cells exposed to microgravity is crucial to eliminate the possibility that microgravity exerts a disruptive effect on cells. The quasi-stability condition would be mirrored by the observation of near-to-unity autocorrelation values across different times for gene expression profiles in the three experimental conditions: OG (cells On Ground), RPMAD (Adherent Cells obtained in simulated microgravity by Random Positioning Machine) and RPMCLUM (Random Machine Positioning Clump cells). We expect that time spent in microgravity only provokes a mild decay from unity correlation with t0, and in any

case, a decline of the same order of magnitude of what happens in OG condition, only due to the forcedly artificial character of *in vitro* cell cultures.

2. We thus posit that the dramatic changes on both morphology and functions (apoptosis, proliferation) observed in cell exposed to microgravity are adaptive modifications, involving only modest changes in the overall gene expression patterns. In this case, quantitative gene changes must be 'buffered' and coordinated across the different genes, so provoking a shift of the system to another sub-attractor state (a new stable situation). This discrete minor (i.e. relative to the minor ruggedness on the bottom of minimum energy valley) 'transition' allows the system in preserving its 'identity' (exactly in the same way the haemoglobin molecule preserves its identity going from R to T configuration).

3. According to this framework, the recovery rate of the native OG gene profile from different physiological states elicited by the microgravity condition is expected to be inversely proportional to the distance of these states from the native OG state. The comeback trajectory should then display a hysteresis-like behavior. Hysteresis means that a system has more than one stable state in respect to changes occurring in a (internal/external) control parameter, where the 'manipulation' of that parameter can eventually enable the reversion to the native state. In this case, the forward and return trajectories are not coincident, due to both the 'memory' (12) of the previous visited states and the non-ideal character of the transformation. Broadly speaking, this means that to induce a switch back to the original stable state (the native 'attractor'), the system needs to go back through another bifurcation point. Such

hysteresis cycles have been largely investigated in ecology (13) but are actually observed also in cell state changes dynamics (14).

We checked point 1) by calculating the autocorrelation in time of the three OG, RPMAD and RPMCLUM conditions. We investigated point 2) by computing the correlation between Euclidean and Angular distances from reference OG state. Euclidean distances embed both additive (expression differences over the entire set of genes) and profile (relative proportions) changes. The Euclidean distance measures how the expression gene profiles differ in magnitude among the different samples, e.g. to what extent the same genes are differently expressed (Supplementary figure 2). On the contrary, cosine (angular) distances correspond to the angle between two vectors. Pearson correlation coefficient permits to measure such distance as it corresponds to the cosine of the angle between the two analysed variable vectors (in our case the gene expression profiles). The angle distance distinguishes how gene expression pattern differs in terms of relation among different gene expression, thus recognizing a *qualitative* difference in their expression pattern. In practice, if we have three genes with expression values 10, 20, 30 in sample A and 20, 40, 60 in sample B, they have non-zero Euclidean distance (d(AB) = 37.4). On the contrary, they have a zero angular distance (the two A and B vectors have a Pearson correlation r = 1, given the proportions among their components do not differ), being Pearson r the cosine of the angle between the two vectors that is to say the two vectors are parallel (zero angle) (15).

The demonstration of a statistically significant and relevant co-variation between Euclidean and Angular measures would demonstrate that the quantitative changes happen in a coordinated manner over the entire set of genes pointing to the need of adjusting mutual gene expression proportions according to the existence of sub-attractors correspondent to different populations. Finally, point 3) was investigated by the projection of different samples on a polar plot where y-axis is the Euclidean distance values and x-axis reports Angle values, determined in respect to the reference OG profile located at the centre of the plot. This representation highlights the hysteresis character of the phenotypic states trajectory with respect to the OG baseline condition, while allowing us to estimate the mutual distances in the gene expression multidimensional phase space among different samples (16).

Supplementary figure 1

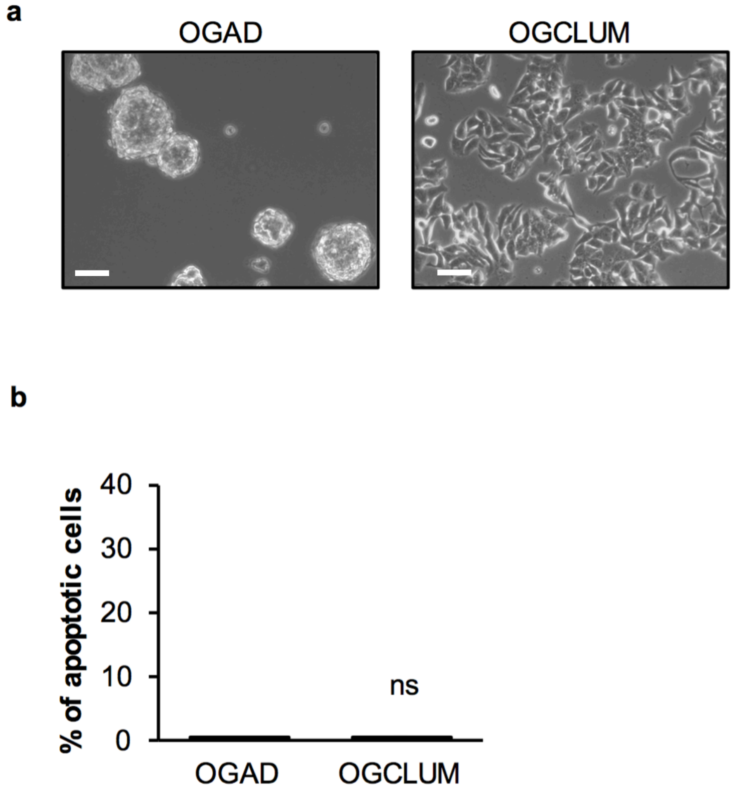

Supplementary figure 1. (a) MCF7 were grown for 72 hours in ultra-low attachment vessels (Corning) to prevent the attachment and the monolayer growth observed in standard OG conditions (OGAD : on ground, adherent ; OGCLUM : on ground, clumps). **(b)** apoptosis was assessed in MCF7 grown in monolayer (OGAD) and in MCF7 grown in non-adherent conditions (OGCLUM). Scale bar : 50 μm. Ns : not significant.

Supplementary figure 2

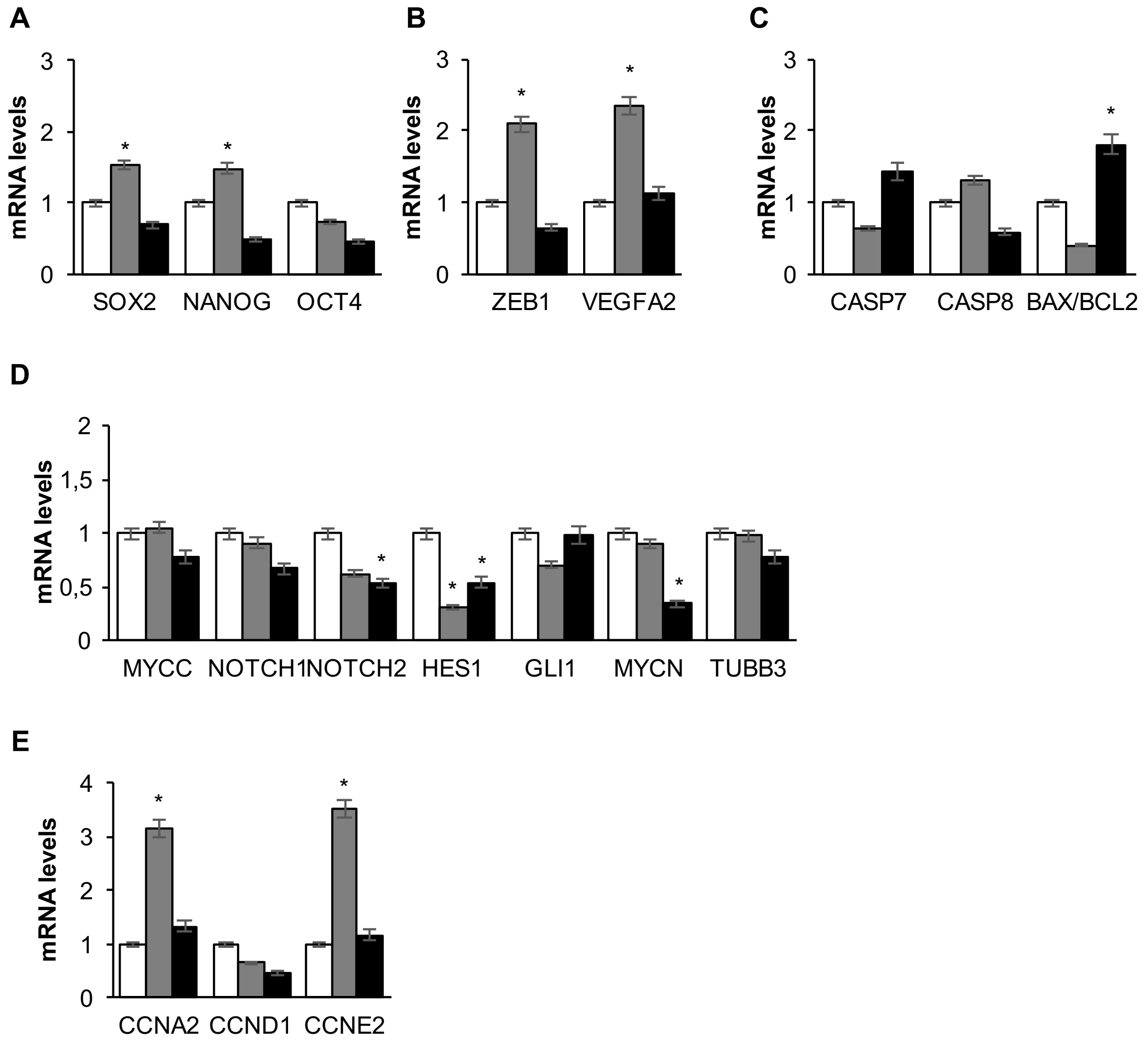

Supplementary figure 2. Histograms show the modulation of analyzed genes in normal gravity (OG) and after microgravity, dividing the two population of adherent cells (RPMAD) and clumps (RPMCLUM). Genes were divided according to pathways: a) reprogramming b) epithelial – mesenchymal transition c) apoptosis d) multidrug resistance, Hedgehog pathway and Notch pathway e) cell cycle. GAPDH, HPRT, beta ACTIN and beta 2 MICROGLOBULIN were used as housekeeping. *$p<0.05$.

Supplementary figure 3

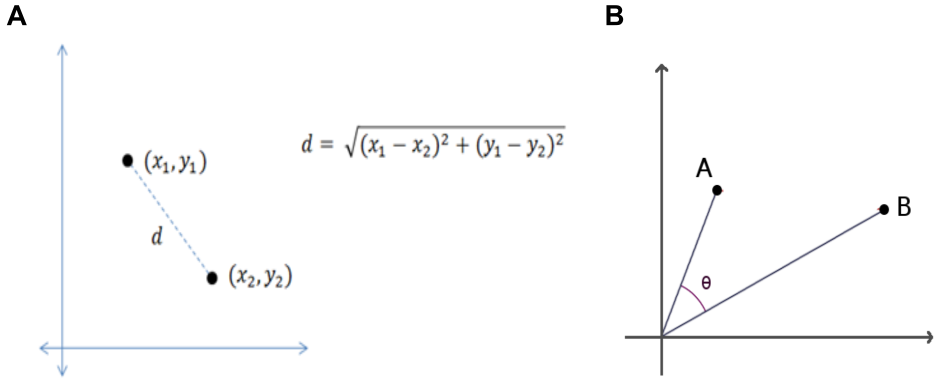

Supplementary figure 3. Metric spaces. We define as 'metric' a space where we can we can compute a distance between any two p and q vectors pertaining to that space. A distance function is defined by three basic properties:

1. d(p, q) ≥ 0 (non-negativity)
2. d(p, q) = 0 if and only if p = q (identity of indiscernibles)
3. d(p, q) = d(q, p) (symmetry)
4. d(p, z) ≤ d(p, q) + d(q, z) (subadditivity / triangle inequality).

In this paper, we deal with two different metrics: Euclidean and Angular (Correlation) metrics.

The following figure reports (dashed line) the Euclidean distance d between two p and q point vectors of coordinate (x1,y1) and (x2,y2). The formula is an application of Pythagoras theorem, being d the hypotenuse of the triangle having cathects: (x1-x2) and (y1-y2) respectively.

The above formula extends to spaces having a number n of dimensions greater than two as (Fig. S1a):

$d(p,q) = \sqrt{\sum_{i=1}^{n}(pi - qi)^2}$

The above formula gives the Euclidean distances between two p and q gene expression profiles with a maximal n = 26 components (different gene expression). In order to cope with missing values, we normalized the above distance formula by dividing it by n(e) = number of gene expressions values actually present in both profiles:

$$dnorm(p,q) = d(p,q)/n(e)$$

This normalization was necessary because any dimension (gene expression value) adds a positive contribution to distance, the distance computation over (slightly) varying dimensionality spaces does not alter the global metrics (17).

While both 'size' and 'shape' differences enter into Euclidean metrics, Correlation metrics takes into consideration only 'shape' differences between two vectors. In other words two p = (10,20,30) and q = (1,2,3) vectors have a null angular distance because the ratios among their components are conserved. In geometrical terms, that is to say we consider the distance between two vectors as the width of the angle between them (Fig. S1b). The cosine of the angles between two vectors correspond to their Pearson correlation coefficient according to:

$$similarity\ (p, q) = \cos(\theta) = \frac{P.Q}{||P||||Q||} = Pearson\ r(p, q)$$

The maximal similarity being correspondent to a unit Pearson correlation between p and q and consequently to $\theta = 0$.

The angular distance is thus

$$Angular\ Distance\ (p, q) = arcos(similarity(p, q) = arcos(r(p, q))$$

In order to go more in depth with these arguments we suggest to read Chapter 6 of:

McCune, B., Grace, J. B., & Urban, D. L. (2002). Analysis of ecological communities. Gleneden Beach.

(https://www.umass.edu/landeco/teaching/multivariate/readings/McCune.and.Grace.2002.chapter6.pdf).

Supplementary table 1

	GAPDH	HPRT	BACTIN	B2 MICRO	SOX2	NANOG	OCT4	ZEB1	VEGFA2	CASP7	CASP8	BAX	BCL2
OG 1h	19,728	27,481	21,978	22,607	23,146	27,992	28,081	32,629	25,245	25,352	25,562	23,241	28,311
OG 2h	18,559	26,650	21,134	21,658	21,933	27,346	27,489	31,445	23,962	24,364	24,385	22,368	27,275
OG 6h	19,430	27,416	21,526	22,230	22,946	28,604	28,381	32,345	24,836	25,518	24,979	22,872	29,280
OG 24h	19,105	27,733	22,271	22,176	22,342	27,510	27,854	31,587	25,138	25,910	24,128	22,823	27,362
RPMAD 1h	18,605	26,667	21,205	22,211	22,709	27,527	28,010	31,897	24,367	25,304	24,738	22,487	27,974
RPMAD 2h	16,955	25,217	20,368	20,645	21,175	26,213	26,713	31,587	22,611	24,082	22,822	21,822	26,629
RPMAD 6h	19,939	27,400	21,625	22,713	23,372	28,794	28,930	32,365	24,952	25,790	25,232	23,500	29,134
RPMAD 24h	15,730	24,481	20,727	20,602	19,537	24,761	26,116	28,336	21,716	24,402	21,545	21,403	24,628
RPMCLUM 1h	19,211	27,058	21,279	22,533	23,398	28,005	28,482	32,401	24,694	25,085	25,182	22,465	28,242
RPMCLUM 2h	20,879	28,821	22,467	24,120	24,540	29,811	30,272	34,188	26,209	27,231	26,773	23,850	29,356
RPMCLUM 6h	18,511	26,685	20,728	21,733	22,323	28,669	28,291	31,592	24,418	24,334	24,250	22,821	28,732
RPMCLUM 24h	18,626	26,631	21,254	21,873	22,185	27,846	28,319	31,516	24,294	24,701	24,210	22,518	27,912
RPMAD 24h + OG 6h	18,938	22,654	17,146	21,926	22,887	27,432	27,889	32,587	25,684	24,645	24,322	22,512	28,568
RPMCLUM 24h + OG 6h	32,648	34,007	29,612	33,595	34,469	36,944	39,050	32,587	35,154	34,496	35,214	33,192	Undet
RPMAD 24h + RPMAD 6h	20,234	23,703	18,631	23,075	24,155	29,193	31,261	28,766	27,101	25,632	25,401	23,402	29,372
RPMCLUM 24h + RPMAD 6h	38,501	Undet	36,187	38,689	Undet	36,980	Undet	32,348	38,202	Undet	Undet	Undet	38,229
RPMAD 24h + RPMCLUM 6h	21,993	25,209	19,982	24,820	26,088	31,089	31,877	32,976	27,532	27,076	26,720	24,351	30,339
RPMCLUM 24h + RPMCLUM 6h	31,284	33,143	29,381	32,987	33,967	35,912	39,443	33,984	32,693	34,923	36,689	31,189	Undet

	MYCC	NOTCH1	NOTCH2	HES1	GLI1	MYCN	TUBB3	CCNA2	CCND1	CCNE2	EFGR	ERBB2	IGFR1
OG 1h	23,316	25,263	22,202	25,675	32,237	31,576	25,229	25,266	21,859	24,371	27,800	25,151	38,317
OG 2h	22,505	24,638	21,247	24,965	32,432	30,115	23,900	24,250	21,049	23,324	27,194	24,209	35,882
OG 6h	23,499	25,255	22,391	25,771	33,439	30,631	24,249	24,395	21,697	24,436	28,458	25,004	37,709
OG 24h	23,071	25,387	21,770	25,022	32,264	29,918	24,464	25,544	21,131	24,391	26,985	23,929	35,153
RPMAD 1h	21,679	24,721	21,637	24,232	31,605	30,221	24,093	24,307	21,194	23,576	27,673	24,670	36,598
RPMAD 2h	21,223	23,739	20,248	23,740	31,304	28,637	23,923	23,499	19,750	21,983	25,711	23,509	34,348
RPMAD 6h	23,673	25,722	22,708	26,183	34,378	31,649	24,459	24,945	22,192	24,585	27,990	25,388	Undet
RPMAD 24h	20,816	23,345	20,265	24,541	30,594	27,881	22,322	21,699	19,566	20,392	24,403	23,410	34,533
RPMCLUM 1h	21,762	24,799	22,211	24,162	32,694	31,384	23,425	23,722	21,374	24,528	28,357	24,924	38,071
RPMCLUM 2h	23,396	25,772	23,492	25,458	33,909	33,283	24,312	24,427	22,776	26,503	29,717	26,452	Undet
RPMCLUM 6h	22,643	24,997	22,100	24,801	32,563	31,480	23,207	23,831	21,350	23,990	27,664	24,651	38,704
RPMCLUM 24h	22,748	25,277	22,013	25,221	31,596	30,767	24,136	24,440	21,544	23,471	27,733	24,504	37,178
RPMAD 24h + OG 6h	20,605	24,631	21,934	25,868	33,003	29,705	25,016	23,764	21,155	23,566	27,725	24,368	20,208
RPMCLUM 24h + OG 6h	23,305	35,560	31,932	37,773	38,108	Undet	35,203	34,543	31,596	35,410	Undet	34,962	30,123
RPMAD 24h + RPMAD 6h	21,816	26,046	23,306	26,899	33,924	30,535	25,797	24,584	22,380	24,698	28,816	25,614	21,424
RPMCLUM 24h + RPMAD 6h	22,031	Undet	36,667	Undet	36,385	Undet	Undet	Undet	37,887	Undet	38,026	Undet	35,611
RPMAD 24h + RPMCLUM 6h	21,816	27,488	24,939	27,379	36,427	32,156	26,714	25,189	23,692	27,107	31,118	27,355	22,835
RPMCLUM 24h + RPMCLUM 6h	22,836	34,592	32,653	32,307	35,974	Undet	34,986	33,507	31,267	35,914	37,126	34,695	30,537

Supplementary table 1. Raw data for gene expression of MCF7 cells exposed to microgravity. Values represent the average of the triplicate as described in material and methods.

Supplementary Table 2

GENE NAME	ID ASSAY
glyceraldehyde-3-phosphate dehydrogenase (GAPDH)	Hs02786624_g1
hypoxanthine guanine phosphoribosyl transferase (HPRT)	Hs02800695_m1
β–Actin (BACTIN)	Hs01060665_g1
β-2-microglobulin (B2MICRO)	Hs00187842_m1
NOTCH1	Hs01062014_m1
NOTCH2	Hs01050702_m1
GLI1	Hs00171790_m1
B-cell lymphoma 2 (BCL2)	Hs00699441_m1
BAX	Hs00180269_m1
Vascular endothelial growth factor A (VEGFA2)	Hs00900055_m1
Cyclin A2 (CCNA2)	Hs00996788_m1
Cyclin D1 (CCND1)	Hs01050839_m1
Cyclin E2 (CCNE2)	Hs00180319_m1
MYC proto-oncogene	Hs00153408_m1
MYCN proto-oncogene	Hs00232074_m1
Caspase7 (CASP7)	Hs00169152_m1
Caspase8 (CASP8)	Hs06630780_s1
epidermal growth factor receptor (EGFR)	Hs01076090_m1
erb-b2 receptor tyrosine kinase 2 (ERBB2)	Hs01001580_m1
insulin like growth factor 1 receptor (IGF1R)	Hs00609566_m1
HES1	Hs00172878_m1
POU Class 5 Homeobox 1 (OCT4)	Hs04260367_gH

SRY-box 2 (SOX2)	Hs01053049_s1
Nanog homeobox (NANOG)	Hs02387400_g1
zinc finger E-box binding homeobox 1 (ZEB1)	Hs01566408_m1
β-3-tubulin (TUBB3)	Hs00801390_s1

Supplementary Table 2. List of Taqman assay IDs for gene expression analyses

Supplementary References

1. Giuliani A, Tsuchiya M, Yoshikawa K. Self-organization of genome expression from embryo to terminal cell fate: single-cell statistical mechanics of biological regulation. Entropy. 2017;20(1):13.
2. Bak P. How nature works: the science of self-organized criticality: Springer Science & Business Media; 2013.
3. Tsuchiya M, Giuliani A, Hashimoto M, Erenpreisa J, Yoshikawa K. Self-organizing global gene expression regulated through criticality: mechanism of the cell-fate change. PloS one. 2016;11(12):e0167912.
4. Ye Z, Sarkar CA. Towards a quantitative understanding of cell identity. Trends in cell biology. 2018.
5. Mojtahedi M, Skupin A, Zhou J, Castano IG, Leong-Quong RY, Chang H, et al. Cell fate decision as high-dimensional critical state transition. PLoS biology. 2016;14(12):e2000640.
6. Zhou JX, Isik Z, Xiao C, Rubin I, Kauffman SA, Schroeder M, et al. Systematic drug perturbations on cancer cells reveal diverse exit paths from proliferative state. Oncotarget. 2016;7(7):7415.
7. Frauenfelder H, Sligar SG, Wolynes PG. The energy landscapes and motions of proteins. Science. 1991;254(5038):1598-603.
8. Giuliani A. Statistical mechanics of gene expression networks: Increasing connectivity as a response to stressful condition. Adv Syst Bio. 2014;3:3-6.
9. Huang S. Cell lineage determination in state space: a systems view brings flexibility to dogmatic canonical rules. PLoS biology. 2010;8(5):e1000380.

10. Censi F, Giuliani A, Bartolini P, Calcagnini G. A multiscale graph theoretical approach to gene regulation networks: a case study in atrial fibrillation. IEEE Transactions on Biomedical Engineering. 2011;58(10):2943-6.
11. Tsuchiya M, Selvarajoo K, Piras V, Tomita M, Giuliani A. Local and global responses in complex gene regulation networks. Physica A: Statistical Mechanics and its Applications. 2009;388(8):1738-46.
12. Harfe BD, Scherz PJ, Nissim S, Tian H, McMahon AP, Tabin CJ. Evidence for an expansion-based temporal Shh gradient in specifying vertebrate digit identities. Cell. 2004;118(4):517-28.
13. Scheffer M, Carpenter SR. Catastrophic regime shifts in ecosystems: linking theory to observation. Trends in ecology & evolution. 2003;18(12):648-56.
14. La Manno G, Soldatov R, Zeisel A, Braun E, Hochgerner H, Petukhov V, et al. RNA velocity of single cells. Nature. 2018;560(7719):494.
15. Johnson RA, Wichern DW. Multivariate analysis. Encyclopedia of Statistics in Quality and Reliability. 2008;3.
16. Reinberg A, Smolensky MH. Investigative methodology for chronobiology. Biological Rhythms and Medicine: Springer; 1983. p. 23-46.
17. Sneath P. Distortions of taxonomic structure from incomplete data on a restricted set of reference strains. Microbiology. 1983;129(4):1045-73.
